# Weak Fault Feature Extraction of Rolling Bearings Based on Adaptive Variational Modal Decomposition and Multiscale Fuzzy Entropy

**DOI:** 10.3390/s22124504

**Published:** 2022-06-14

**Authors:** Zhongliang Lv, Senping Han, Linhao Peng, Lin Yang, Yujiang Cao

**Affiliations:** College of Mechanical and Power Engineering, Chongqing University of Science and Technology, Chongqing 401331, China; 2020203034@cqust.edu.cn (S.H.); 2021203215@cqust.edu.cn (L.P.); 2020203030@cqust.edu.cn (L.Y.); 2021203230@cqust.edu.cn (Y.C.)

**Keywords:** Adaptive Variational Modal Decomposition, correlation coefficient, Multiscale Fuzzy Entropy, feature extraction, Particle Swarm Optimization

## Abstract

The working environment of rotating machines is complex, and their key components are prone to failure. The early fault diagnosis of rolling bearings is of great significance; however, extracting the single scale fault feature of the early weak fault of rolling bearings is not enough to fully characterize the fault feature information of a weak signal. Therefore, aiming at the problem that the early fault feature information of rolling bearings in a complex environment is weak and the important parameters of Variational Modal Decomposition (VMD) depend on engineering experience, a fault feature extraction method based on the combination of Adaptive Variational Modal Decomposition (AVMD) and optimized Multiscale Fuzzy Entropy (MFE) is proposed in this study. Firstly, the correlation coefficient is used to calculate the correlation between the modal components decomposed by VMD and the original signal, and the threshold of the correlation coefficient is set to optimize the selection of the modal number K. Secondly, taking Skewness (Ske) as the objective function, the parameters of MFE embedding dimension M, scale factor S and time delay T are optimized by the Particle Swarm Optimization (PSO) algorithm. Using optimized MFE to calculate the modal components obtained by AVMD, the MFE feature vector of each frequency band is obtained, and the MFE feature set is constructed. Finally, the simulation signals are used to verify the effectiveness of the Adaptive Variational Modal Decomposition, and the Drivetrain Dynamics Simulator (DDS) are used to complete the comparison test between the proposed method and the traditional method. The experimental results show that this method can effectively extract the fault features of rolling bearings in multiple frequency bands, characterize more weak fault information, and has higher fault diagnosis accuracy.

## 1. Introduction

As the rotating support components of most machinery, the fault detection and diagnosis of rotating machinery such as rolling bearings is essential to prevent mechanical failures [1,2]. A variety of bearing fault detection techniques such as acoustic emission, electrostatic and vibration are used meticulously by industrial enterprises [3,4,5]. Among them, vibration monitoring is the most established diagnostic technique for rolling element bearing. To ensure the smooth operation of the bearing, it is important to study its fault diagnosis method. The operating characteristics of most bearings are unstable, and it is difficult to extract fault features for its large vibration and noise interference. Therefore, accurate fault feature extraction is the key to fault diagnosis of rolling bearings under complex environment interference.

The key to fault diagnosis lies in analyzing the original signals from the time-frequency domain and constructing feature sets from different aspects to describe the running state of the rotating machinery. At present, one of the most commonly used time-frequency analysis methods is Empirical Mode Decomposition (EMD) [6]. Bustos et al. [7] proposed a signal processing method based on EMD in order to monitor the early defects of key components of high-speed trains. However, EMD has problems such as endpoint effect and modal aliasing [8]. Ensemble Empirical Mode Decomposition (EEMD) is an improved algorithm for EMD, which can effectively suppress mode aliasing [9]. Local Mean Decomposition (LMD) [10] improves the problem of overdevelopment or underdevelopment in EMD, but its essence, similar to EMD and EEMD, belongs to recursive mode decomposition, and ultimately cannot avoid the endpoint effect and mode aliasing. Dragomiretskiy and Zosso proposed a non-recursive modal decomposition method, namely VMD, according to the constrained variational problem, which can effectively reduce the occurrence of modal aliasing [11]. Li et al. [12] optimized VMD parameters based on the principle of minimum information entropy, and then obtained the Intrinsic Mode Function (IMF) of minimum information entropy as the effective IMF component for envelope demodulation analysis to extract bearing fault features. Zheng et al. [13] proposed a signal feature extraction method based on VMD and Permutation Entropy (PE), which utilized Permutation Entropy to construct fault feature sets for fault identification and achieved good results. Xia et al. [14] proposed a method for combining Maximum Correlated Kurtosis Deconvolution (MCKD) and VMD to extract rolling bearing fault features. The maximal correlation kurtosis deconvolution is used to enhance the fault features, and the kurtosis criterion cross-relation number is used to reconstruct the signal to extract the fault features with rich fault information.

Fuzzy Entropy (FE) is a measure of the probability that a time series will generate new patterns when its dimensionality changes [15]. Moreover, the greater the probability of the sequence generating new patterns, the greater the complexity of the sequence and the greater the entropy value will be. In 2014, Zhen et al. [16] proposed a new method for measuring the complexity of time series: Multiscale Fuzzy Entropy (MFE), which represents the complexity and self-similarity of time series under different scale factors. Therefore, a fault diagnosis method of rolling bearings based on MFE and Support Vector Machine (SVM) [17] is proposed. Although MFE contains temporal pattern information on different scales and reflects the inherent characteristics of the signal, the performance of signals with similar characteristics is not ideal. Li et al. [18] proposed a new method to reflect signal complexity or nonlinearity based on MFE. Li used the skewed distribution characteristics of fuzzy entropy values at different scales to quantitatively characterize the complexity or nonlinearity of the signal, and more accurately reflect the characteristics of the signal. In order to accurately use vibration signals for fault diagnosis, Yang et al. [19] proposed a gear fault diagnosis method based on EEMD-MFE. This method utilizes the fuzzy entropy of the IMF component obtained by EEMD decomposition and takes the fuzzy entropy of the original signal at multiple scales as the characteristic parameter of different gear states. Fan et al. [20] decomposed the sample signal by VMD in order to realize the sound recognition of different models under different working conditions. MFE calculation is performed on the obtained intrinsic mode functions of different scales, and MFE features are obtained. Xu et al. [21] proposed a fault diagnosis model for rolling bearings that combines fine composite MFE and a Particle Swarm Optimization support vector machine. Compared with fine composite Multiscale Sample Entropy (MSE) and MFE, the smoothness of the fine composites MFE model is better. For the problem that the characteristic information of rolling bearings is difficult to effectively extract under the influence of a harsh environment, Ding et al. [22] proposed a method based on Local Mean Decomposition (LMD) and MFE. The rolling bearing fault diagnosis algorithm first used LMD to decompose the bearing vibration signal, and then extracted MFE features for each component.

Most of the above methods only consider the fault diagnosis in the case of a single feature, which creates a problem of insufficient fault feature representation. In order to describe the weak fault features from multiple perspectives better, this study proposes an early fault feature extraction method that combines AVMD and MFE. The parameters of the MFE are optimized by the Particle Swarm Optimization (PSO) [23] algorithm, so that the extracted MFE features can characterize the fault information to the greatest extent. Taking Ske as the objective function, the parameters of MFE embedding dimension M, scale factor S and time delay T are optimized by PSO algorithm. The MFE eigenvectors of each frequency band after AVMD are obtained. After the AVMD, the modal components in each frequency band of the original signal are better separated, and the MFE after their parameter optimization is extracted. The method can extract the optimized MFE in multiple frequency bands of the fault signal and characterize more weak fault information. It is more beneficial to identify the early weak faults of rolling bearings.

The second part of the article introduces the principles of AVMD and PSO to optimize MFE. The third part introduces the specific process of constructing a fault feature set based on AVMD combined with optimized MFE. The fourth part uses the DDS test bench for experimental analysis. It is proved that the combination of AVMD and optimized MFE can describe the MFE characteristics of fault signals in multiple frequency bands, which can better characterize more weak fault information, and is more sensitive to the early weak faults of rotating machinery. Additionally, compared with the traditional decomposition method, the method proposed in this study has higher fault diagnosis accuracy.

## 2. Basic Theory

### 2.1. Variational Modal Decomposition

The overall framework of VMD is to solve the variational problem in order to minimize the sum of the estimated bandwidths of each eigenmodal function, where each eigenmodal function is assumed to be a finite bandwidth with different center frequencies. To solve this variational problem, the alternating direction multiplier method is used to continuously update each eigenmode function and its center frequency, which can demodulate each eigenmode function to the corresponding fundamental frequency band and, finally, extract each eigenmode function and its corresponding center frequency.

The decomposition steps of the variational modal decomposition are as follows:

(1) Initialize $\left\{u_{k}^{1}\right\},\left\{\omega_{k}^{1}\right\}$, $\lambda_{k}^{1}$ and n=1;
(2) n=n+1, entering the loop;
(3) update according to the update formula for $u_{k}$ and $\omega_{k}$ until the inner loop stops when the number of decompositions K is reached;
(4) update λ according to the update formula of λ;
(5) Given the precision ω, if the stopping condition ∑k||ukn+1−ukn||22||ukn||22<ε, is satisfied, stop the loop, otherwise enter step 2 to continue the loop.

Where $u_{k}$ is the decomposed unit-component IMF signal; $\omega_{k}$ is the center frequency of each but component IMF signal; λ is the Lagrange multiplier; n is the number of iterations.

### 2.2. Adaptive Variational Modal Decomposition

Compared with EEMD and other adaptive decomposition algorithms, the VMD algorithm has better sparse modal components, but the decomposition result of the VMD algorithm is affected by multiple parameters, among which the modal number K and penalty factor α have a great impact on the VMD algorithm. Improper parameter selection will cause over-decomposition, modal aliasing and false modes. Therefore, the parameter optimization of modal number K and penalty factor α is the focus of scholars’ discussion.

This article chooses the method of combining the correlation coefficient with VMD to optimize the value of parameter K in VMD. The correlation coefficient method is one of the most important methods to process and analyze signals. In the segmentation method proposed in this study, the correlation coefficient is used to calculate the correlation coefficient between the modal component obtained after VMD and the original signal. The mathematical model of the correlation coefficient is as follows.

$$\rho_{xy}=\frac{\sum_{i=1}^{N}\left(x_{i}-\bar{x}\right)\left(y_{i}-\bar{y}\right)}{\sqrt{\sum_{i=1}^{N}{\left(x_{i}-\bar{x}\right)}^{2}}\sqrt{\sum_{i=1}^{N}{\left(y_{i}-\bar{y}\right)}^{2}}}$$

In the formula, $\bar{x}$ is the mean value of the IMF component, $\bar{y}$ is the mean value of the original signal and $\left|\rho_{xy}\right|$ is the correlation coefficient of the two sets of signals, the range is between [−1,1]. The weaker the correlation, the closer $\left|\rho_{xy}\right|$ gets to 0, indicating that the component signal has little or no correlation with the original signal.

Therefore, due to the smaller $\left|\rho_{xy}\right|$, this study chooses to set a correlation coefficient threshold as the critical value of the correlation coefficient in the proposed AVMD algorithm and use it to optimize the value of the modal number K.

The penalty factor is one of the parameters that must be adjusted manually in VMD. Too small a penalty factor α will increase the probability of mode aliasing and too large a penalty factor α will weaken the effect of noise reduction. According to the spectral distribution characteristics of the fault vibration signal of rotating machinery, the mid-low frequency region is mainly composed of the harmonics of the rotating frequency and its related characteristic frequencies (such as the bearing fault characteristic frequency and gear meshing frequency, etc.), while the fault impact and noise interference are mostly located in the high frequency area. At the same time, the harmonic signal has the characteristics of longtime domain duration and relatively compact frequency domain, while the impulse signal has the characteristics of a short time domain and wide frequency domain. Therefore, in order to better separate the inherent harmonic signal, fault impact and noise signal, the value of the penalty factor α=1/2fs~2fs is set to verify the decomposition effect of AVMD in this study.

**Figure 1:**
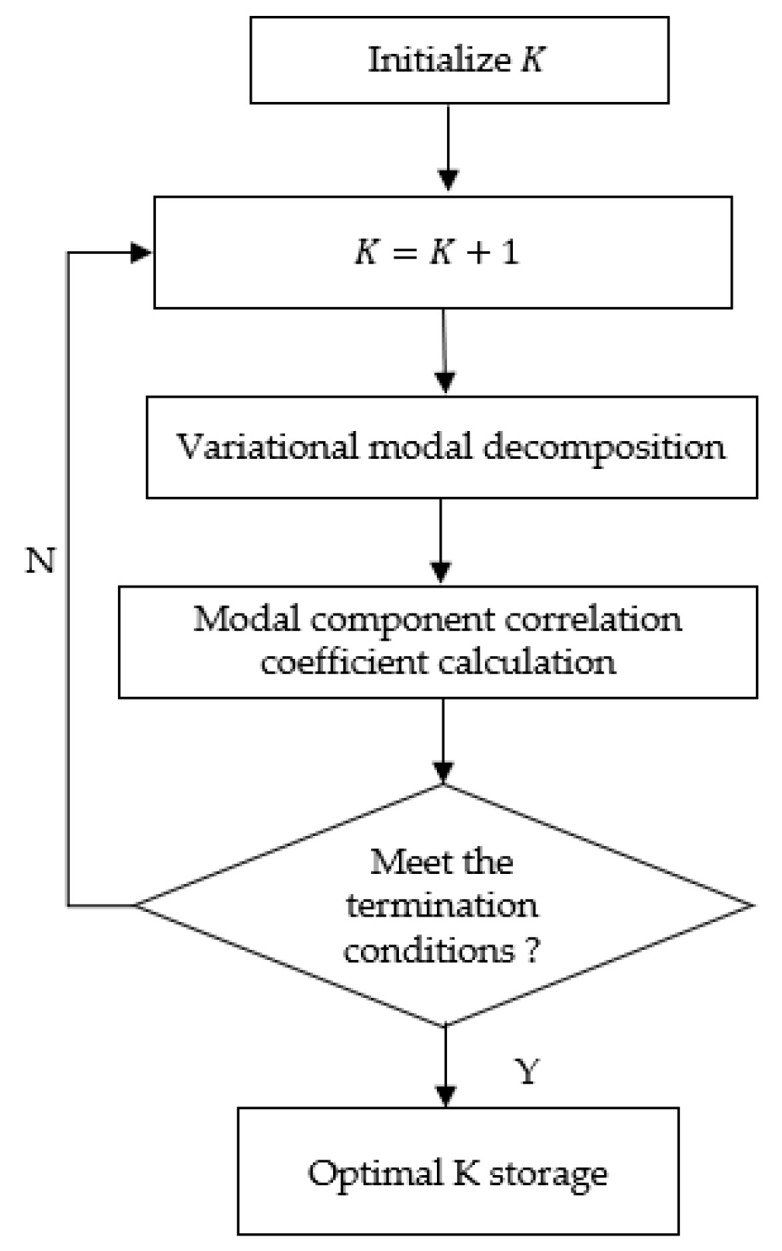
Adaptive Variational Mode Decomposition Process.

VMD and the correlation coefficient are combined to optimize the selection of the modal number. The initial modal number K=2. Perform VMD on the signal and obtain the correlation coefficient between each mode after decomposition and the original signal. If the minimum value of the correlation coefficient between each mode and the original signal after decomposition is less than the threshold, the decomposition will stop. Otherwise, the mode will increase the number and continue to decompose until the stopping condition is met, in order to determine the modulus K, and finally store the optimal value of K. The AVMD flow chart is shown in Figure 1.

In order to check the effectiveness of the algorithm, this section takes the simulated signal as an example, and uses the AVMD algorithm proposed in this article to verify the decomposition effect. For periodic simulation signal: $x\left(t\right)=x_{1}\left(t\right)+x_{2}\left(t\right)+x_{3}\left(t\right)$*,* $x_{1}\left(t\right)=\cos8\pi t,\;x_{2}\left(t\right)=\frac{1}{4}\times \cos96\pi t,x_{3}\left(t\right)=\frac{1}{16}\times \cos576\pi t$, the actual signal contains three modes: cosine with amplitude of 1 at 4 Hz, cosine with amplitude of 1/4 at 48 Hz and cosine with amplitude of 1/16 at 288 Hz. Perform AVMD on it, preset different K (K=2, 3, 4, 5) values, and the penalty factor α=fs. Then calculate the correlation coefficient between each modal component and the original signal under different K values, and the results are shown in Table 1.

**Figure 2:**
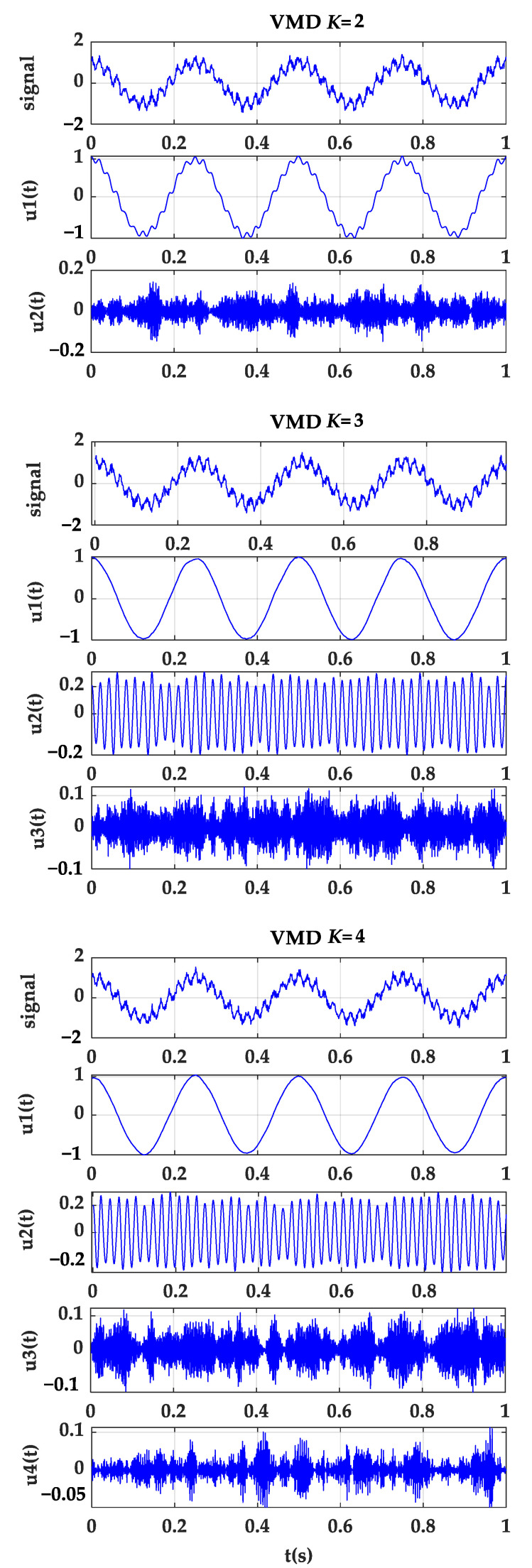
K=2, 3, 4 VMD exploded view.

It can be seen from the correlation coefficient between each modal component and the original signal that when K=2, the correlation coefficient between the modal com-ponent u2 and the original signal is 0.1083. When K=4, the correlation between the modal component u4 and the original signal is 0.1083. The coefficient is only 0.0878 (less than the set threshold E=10%). Then, the selected mode number K=3 meets the iterative stop condition and makes the VMD diagram when K=2, 3, 4, as shown in Figure 2.

As shown in Figure 2, when K=3, the three modes are well separated. However, when K=2, it can be seen that the 4 Hz cosine signal and the 48 Hz cosine signal in the original signal are superimposed together, and the phenomenon of “modal aliasing” appears; when K=4, u1(t), u2(t) represents the cosine signal of 4 Hz and 48 Hz in the original signal, and u3(t) represents the cosine signal of 288 Hz, but a false mode u4(t) also appears.

**Figure 3:**
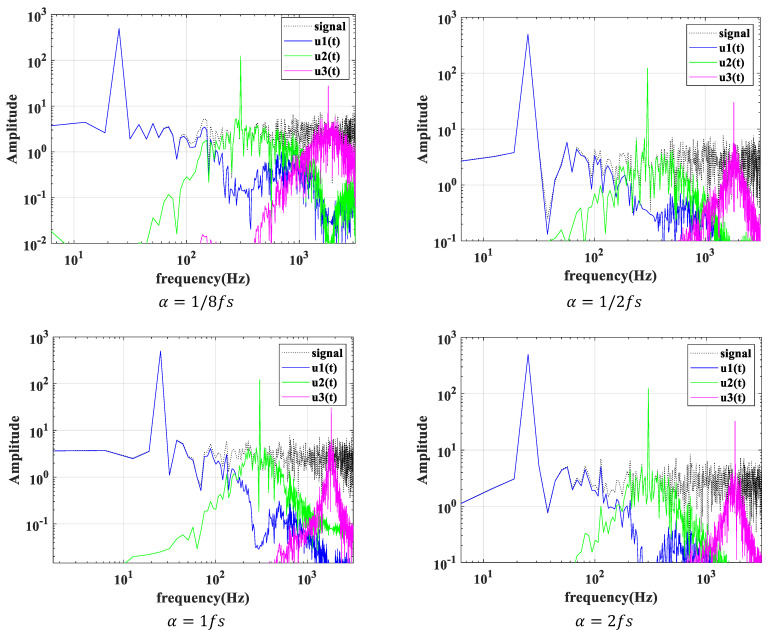
α=1/8fs, 1/2fs, fs, 2fs modal signal spectrum distribution.

According to the sampling frequency of the periodic simulation signal, fs=1000 Hz, take K=3; take α=1/8fs, 1/2fs, fs, 2fs respectively, and make the spectrum of each modal component after VMD under different penalty factor α values distribution map, as shown in Figure 3.

From the spectrogram of each modal component after VMD under different α values, it can be seen that when α=1/8fs, 2fs, each modal signal component experiences a serious modal aliasing phenomenon; when  α=fs, 1/2fs, the phenomenon of component modal aliasing is weakened, but there are still some frequency components that have not been decomposed. This is mainly because the bandwidth of the Wiener filter is narrow at this time, which belongs to some frequencies of the original signal. Components will be filtered, resulting in missing information, and some frequency components will appear in multiple components at the same time.

Therefore, from the above analysis of the simulated signal, it can be seen that the default penalty factor α=1/2fs, fs proposed in this study, and the adaptive variational modal decomposition method selected by optimizing the modal number K through the correlation coefficient, can well separate the signal from high frequency to low frequency and avoid the phenomenon of modal aliasing. The decomposition algorithm has a good effect on the decomposition of the signal.

### 2.3. Multiscale Fuzzy Entropy

Multiscale Fuzzy Entropy (MFE) is improved on the basis of fuzzy entropy and combined with multiscale entropy to measure the complexity and similarity of time series under different scale factors. MFE can be calculated as follows:

(1) Coarse-grained original time series X={x(t),t=1,2,…,N} is processed to obtain coarse-grained sequence $y^{\left(\tau \right)}=\{y_{d}^{\tau },1\leq d\leq N^{\prime }/\tau \}$.

$$y_{d}^{\tau }=\frac{1}{\tau }\overset{d_{\tau }}{\underset{t=\left(d-1\right)\tau +1}{\sum }}x_{t}$$

where τ is a scale factor and is generally a positive integer.

(2) Calculate the fuzzy entropy of coarse-grained sequence under each scale factor, and its calculation formula is:

$$MEF\left(X,\tau ,m,r\right)=FE\left(y^{\left(\tau \right)},m,r\right)$$

where m is the embedding dimension; r is similarity tolerance.

The parameter selection of the MFE has a great influence on the extracted MFE features of the vibration signal. Selecting the default parameter settings cannot adequately characterize the weak fault characteristics of rotating machinery. It will have a greater impact on the diagnostic results.

### 2.4. PSO-Optimized Multiscale Fuzzy Entropy

The Particle Swarm Optimization (PSO) algorithm is one of the evolutionary algorithms. It starts from a random solution, finds the optimal solution through iteration, and evaluates the quality of the solution through fitness. However, it is simpler than the genetic algorithm rule, and finds the global optimum by following the currently searched optimal solution. This algorithm has the advantages of easy implementation, high precision and fast convergence. Additionally, it has demonstrated its superiority in solving practical problems. Therefore, this study chooses to optimize the parameters of MFE with a PSO algorithm.

The PSO is initialized to a group of random particles (random solutions), and then the optimal solution is found through iteration. At each iteration, the particle updates itself by tracking two “extreme values” (pbest,gbest). After finding these two optimal values, the particle updates its velocity and position by using the formula below.

$$v_{i}=v_{i}+c_{1}\times rand()\times \left(pbest_{i}-x_{i}\right)+c_{2}\times rand()\times \left(gbest_{i}-x_{i}\right)$$

In order to improve the performance of the algorithm, the weight ω is introduced to $v_{i}$ of the above formula, as shown in the following formula.

$$v_{i}=\omega \times v_{i}+c_{1}\times rand()\times \left(pbest_{i}-x_{i}\right)+c_{2}\times rand()\times \left(gbest_{i}-x_{i}\right)$$

$$x_{i}=x_{i}+v_{i}$$

The above two formulas form the standard form of the PSO algorithm.

It is necessary to determine a fitness function when a PSO algorithm is used to find the optimal parameter of MFE. At this point, the Ske of the data can be obtained. The larger the absolute Ske is, the more problematic the efficiency of the mean is, or the smaller the absolute Ske is, the more reliable the mean is.

In this study, the square function of Ske of MFE is selected as the objective function to find its minimum value. In this way, the parameter values of the embedding dimension M, the scale factor S and the time delay T of the MFE are optimized.

$$\mathrm{Ske}=E{[H_{p}\left(X\right)-H_{p}^{m}\left(X\right)]}^{3}/{[H_{p}^{d}\left(X\right)]}^{3}$$

where $H_{p}^{m}\left(X\right)$ is the mean value of sequence $H_{p}\left(X\right)$; $H_{p}^{d}\left(X\right)$ is the standard deviation of sequence $H_{p}\left(X\right)$; E is the expectation of finding the sequence.

## 3. Fault Feature Set Construction

The MFE was calculated for the modal components in each frequency band after AVMD, and the MFE feature set was constructed to characterize more weak fault information. The specific construction method of an MFE feature set is as follows.

**Figure 4:**
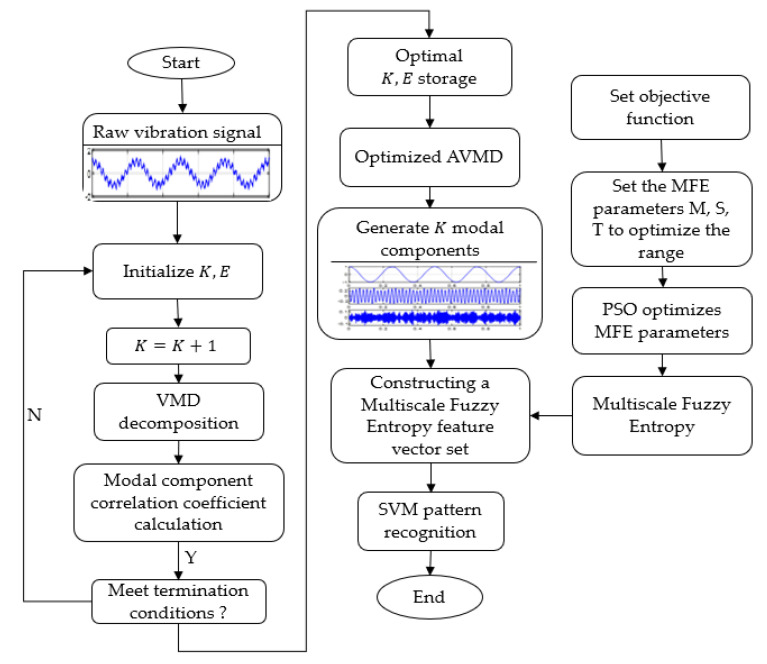
AVMD-based fault diagnosis flow chart.

The specific steps and flowchart of the method based on adaptive variational modal decomposition and fuzzy entropy feature set construction are shown in Figure 4.

(1) Obtain the original signal, initialize the modal number K=2, use the default value of the penalty factor α and the correlation coefficient threshold E: α=fs, E=10%.
(2) Perform VMD on the vibration signal and calculate the correlation coefficient between each mode and the original signal. When the correlation coefficient satisfies the termination condition, the correlation coefficient threshold less than E=10%, and the optimal mode number K and penalty factor α are determined.
(3) The optimized VMD is performed on the vibration signal to generate K modal components.
(4) In order to minimize the Ske of the original signal, the optimal MFE parameters are obtained by adaptive optimization using the PSO algorithm.
(5) Calculate the MFE of K modal components to construct a multiscale and multiband fuzzy entropy feature set.
(6) Input the fuzzy entropy feature set obtained in the previous step into the classifier for fault identification.

## 4. Experimental Verification

**Figure 5:**
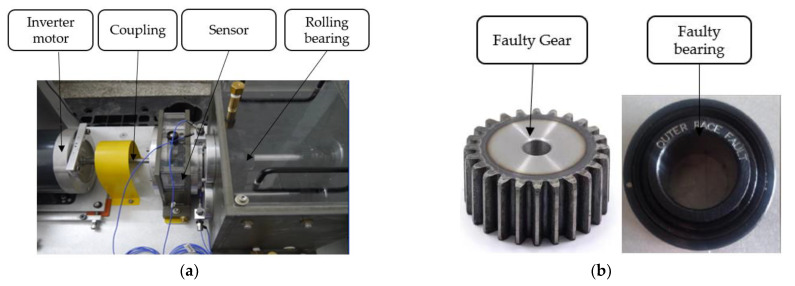
DDS comprehensive test bench and faulty gear. (**a**) DDS comprehensive failure test bench. (**b**) Test bearing.

In order to further verify the effectiveness of the method proposed in this article, five different types of bearing and gear faults on the DDS test bench were collected, and the cube method proposed in this article was used for fault diagnosis to prove the effectiveness of the method. Five different categories of bearing and gear failure are: (1) inner ring fault; (2) outer ring fault; (3) rolling body fault; (4) inner ring + gear wear fault; (5) inner ring + broken tooth fault. The DDS fault diagnosis comprehensive test bench and test gear are shown in Figure 5.

**Figure 6:**
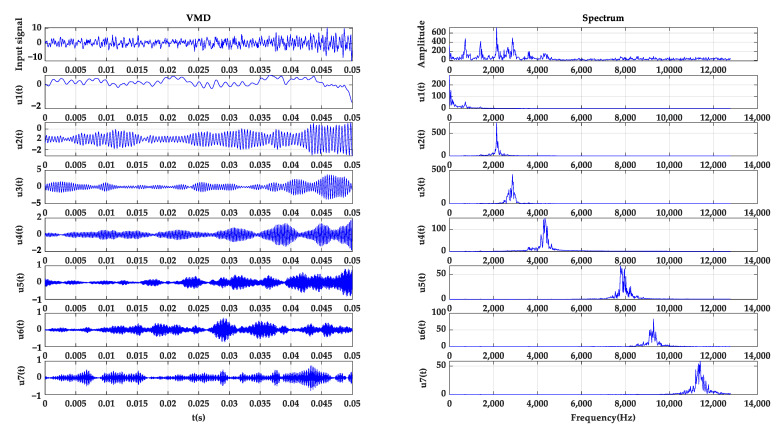
AVMD decomposition and spectrum diagram of inner ring fault signal.

Using the method proposed in this study, the initial AVMD cross-correlation coefficient threshold was set to E=10% and the penalty factor α was set to 1/2fs. AVMD decomposes five different fault signals. Among them, the inner ring signal is decomposed into seven modal components, and the correlation coefficient between each modal component and the original signal is calculated. When K=8 (as shown in Table 2), the cross-correlation coefficient is 0.0947, which exceeds the initial value. The set stop threshold is 10%. The modal components obtained by the AVMD of the outer ring fault, inner ring fault, rolling element fault, gear wear fault and gear broken tooth fault of the signal are shown in Table 3. The original signal of the inner ring fault and the modal component spectrum results after AVMD are shown in Figure 6. It decomposes the inner ring fault signal into seven different modal components from high frequency to low frequency. It can be seen from the spectrogram that the modal components of each frequency band have no modal aliasing phenomenon, which can effectively avoid the phenomenon of modal aliasing. The phenomena of modal aliasing and end-effects that can be caused by traditional adaptive decomposition methods are eliminated.

After that, taking Ske as the objective function, the parameters of MFE embedding dimension M, scale factor S and time delay T are optimized by PSO algorithm. Set the population size to 15 and the maximum iteration times to 30. Set the value range of optimization parameters according to fault signal characteristics: M is [2, 5], S is [1, 10] and T is [1, 5]. The MFE parameters of the five types of fault vibration signals are shown in Table 4.

A total of 500 samples was extracted from five different fault vibration signals collected by the DDS test bench, with each sample having 1024 sampling points. It can be seen from Table 3 that the modal number K value obtained by the inner ring fault after AVMD is the smallest, so extract the first seven modal components of the fault signal decomposed by AVMD. After that, the MFE of each modal component is calculated based on the optimal MFE parameters in Table 4, and the MFE feature set is constructed as shown in Table 5.

**Figure 7:**
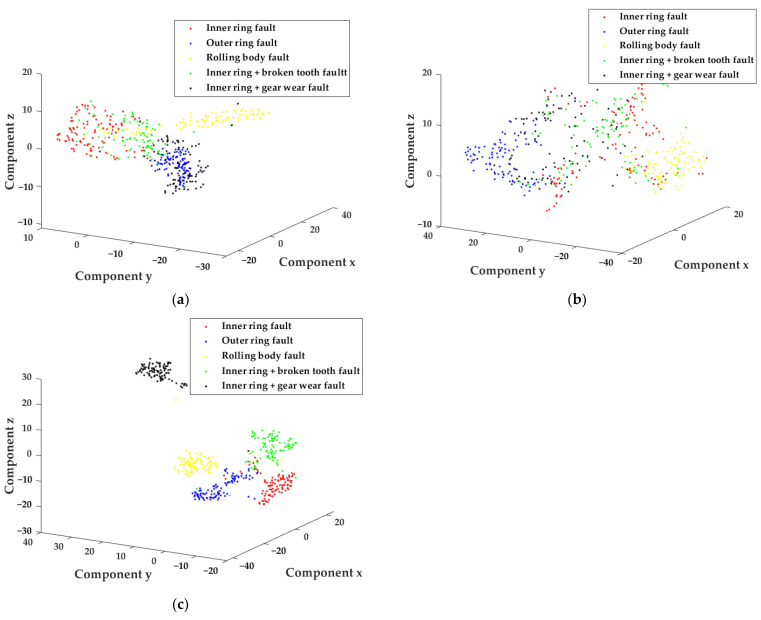
T-SNE dimensional reduction visualization of MFE feature set. (**a**) EEMD-PSO-MFE feature set. (**b**) LMD-PSO-MFE feature set. (**c**) AVMD-PSO-MFE feature set.

**Figure 8:**
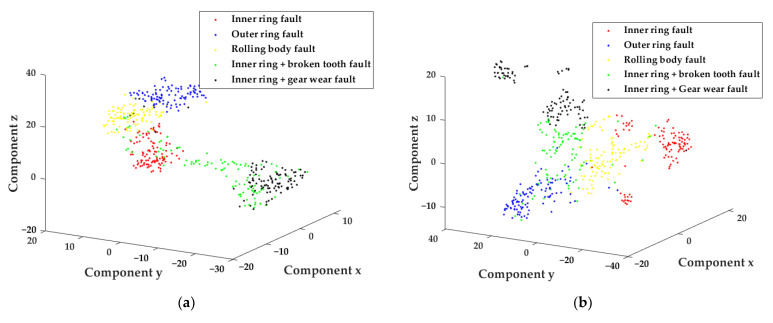

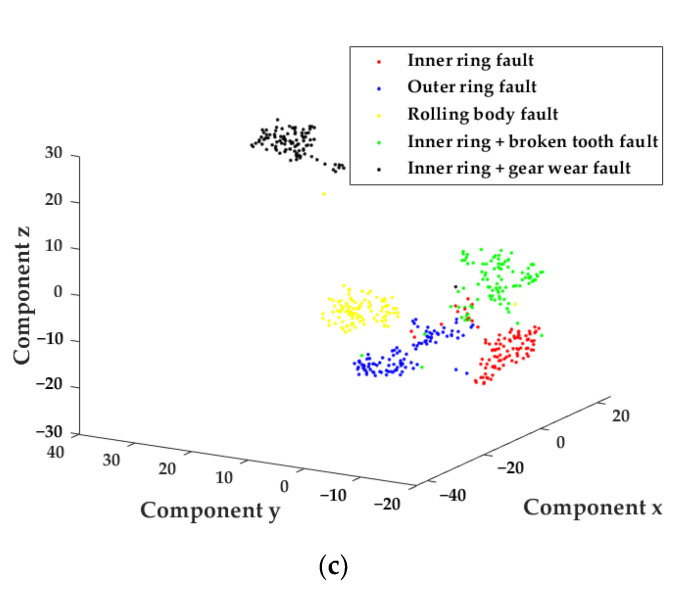
T-SNE dimensionality reduction visualization of FE, MFE and PSO-MFE feature sets. (**a**) AVMD-FE feature set. (**b**) AVMD-MFE feature set. (**c**) AVMD-PSO-MFE feature set.

In order to further analyze the feature vector set constructed by the method in this study, the T-SNE method was introduced to visualize the eigenvector set, and the distribution of five types of faults in the low-dimensional space was observed. As shown in Figure 7 and Figure 8, Figure 7a–c, respectively, shows the distribution of the fault feature set constructed by EEMD, LMD and AVMD combined with the parameter-optimized MFE in the low-dimensional space. Figure 8a–c, respectively, shows the distribution of the fault feature set constructed by combining AVMD with FE, MFE and parameter-optimized MFE in low-dimensional space.

As shown in the Figure 7 and Figure 8, the method proposed in this study can distinguish faults better than the other categories. The MFE feature set constructed in this way can represent more fault information. In addition, it can distinguish fault types in fault diagnosis better and be more sensitive to the weak fault features of rotating machinery.

The MFE feature set constructed by the five methods were put into the SVM, and the accuracy of the five methods is shown in Table 6.

As shown in Table 6 and Figure 7, the method combining AVMD and optimized MFE proposed in this study is compared with the traditional EEMD and LMD signal decomposition methods. AVMD reaches 98% accuracy, which is much higher than the traditional signal decomposition method. It shows that AVMD is superior to EEMD and LMD in signal decomposition.

It can be seen from Table 6 and Figure 8 that the fault feature set is constructed by combining AVMD with FE, MFE and optimized MFE. The fault feature set constructed by combining AVMD and optimized MFE has more obvious differences in the feature distribution of low-dimensional space, indicating that it is more sensitive to the fault features of vibration signals, it can represent more weak fault information and is more conducive to distinguishing fault types. The fault diagnosis accuracy of 98% compared to the other two methods of 89.3% and 81.3% further proves the superiority of the method proposed in this study.

## 5. Conclusions

In this study, a method combining AVMD and optimized MFE is proposed to construct a fault feature set, and this method is verified by DDS test bench data. Compared with other decomposition methods and feature extraction methods, the fault diagnosis accuracy rate in the proposed method reaches the highest 98%. The superiority of the method proposed in this study is proved.

(1) By analyzing the simulation and experimental results, AVMD optimizes the mode number K in VMD by using the correlation coefficient, which can reduce the phenomenon of mode aliasing and excessive decomposition.
(2) The early fault feature extraction method based on AVMD and optimized MFE mainly decomposes the fault signal through AVMD. Taking Ske as the objective function, PSO searches for the optimal parameters of MFE and extracts the MFE features in multiple frequency bands. Through this method, the MFE of the modes in different frequency bands is calculated by decomposing the adaptive variational modes, and the MFE in different frequency bands is used to form a feature vector set. In this way, the weak fault information of rotating machinery can be more fully characterized, and it is more conducive to the early weak fault identification. Simultaneously, it can achieve higher fault diagnosis accuracy.
(3) The MFE feature extraction method based on AVMD can effectively extract the weak fault information of the fault signal, but the calculation amount of MFE is large. The next work will improve this problem to improve the computational efficiency.

## Figures and Tables

**Table 1:** Correlation coefficients between each modal component and the original signal under different K values.

Modulus\Correlation Coefficient	$\rho_{1}$	$\rho_{2}$	$\rho_{3}$	$\rho_{4}$	$\rho_{5}$
K=2	0.9923	0.1038			
K=3	0.9765	0.2564	0.1535		
K=4	0.9813	0.2846	0.1689	0.0878	
K=5	0.9625	0.2987	0.1257	0.0756	0.0878

**Table 2:** Correlation coefficient between each mode and the original signal when the bearing inner ring faults with different K values.

The Modulus\The Correlation Coefficient	$\rho_{1}$	$\rho_{2}$	$\rho_{3}$	$\rho_{4}$	$\rho_{5}$	$\rho_{6}$	$\rho_{7}$	$\rho_{8}$
K=2	0.5576	0.2306						
K=3	0.5638	0.2637	0.2303					
K=4	0.5544	0.5407	0.3163	0.2217				
K=5	0.4042	0.4877	0.5364	0.3247	0.2184			
K=6	0.4036	0.4871	0.5348	0.2108	0.3207	0.2175		
K=7	0.2646	0.3877	0.4737	0.5335	0.2620	0.2827	0.2142	
K=8	0.2578	0.3819	0.4684	0.4627	0.4218	0.3105	0.2180	0.0947

**Table 3:** The number of modes obtained by decomposing different fault signals.

Modal Number	Inner Ring Fault	Outer Ring Fault	Rolling Body Fault	Inner Ring + Gear Wear Fault	Inner Ring + Broken Tooth Fault
K	7	8	8	7	9

**Table 4:** Multiscale Fuzzy Entropy parameters for five kinds of fault.

Parameter\Fault Type	Inner Ring Fault	Outer Ring Fault	Rolling Body Fault	Inner Ring + Gear Wear Fault	Inner Ring + Broken Tooth Fault
M	5	3	5	5	5
S	10	3	4	6	7
T	1	1	4	1	5

**Table 5:** MFE feature sets for different faults.

Fault Type	Sample Number	Feature Vector							Expected Output
Inner ring	1	0.0031	0.1380	0.4990	0.5983	0.7603	0.6467	0.3830	1
	2	0.0036	0.1799	0.4101	0.5768	0.7545	0.7067	0.3755	
	3	0.0033	0.1544	0.4293	0.5907	0.7692	0.7662	0.3346	
Rolling element	10	0.0111	0.1015	0.4513	0.6587	0.413	0.5540	0.2112	2
	102	0.0145	0.1735	0.4338	0.6330	0.4024	0.3927	0.2496	
	103	0.0195	0.1022	0.4587	0.6104	0.4922	0.5042	0.2097	
Outer ring	201	0.0044	0.1459	0.5183	0.5314	0.7753	0.4901	0.3953	3
	202	0.0046	0.1449	0.5966	0.5168	0.7162	0.5975	0.3323	
	203	0.0048	0.1304	0.5736	0.5109	0.7880	0.5312	0.3228	
Inner ring + gear wear	301	0.0085	0.1418	0.5929	0.6776	0.8001	0.7140	0.4273	4
	302	0.0082	0.1216	0.5738	0.6483	0.8968	0.5445	0.4502	
	303	0.0084	0.1808	0.5526	0.6102	0.8114	0.7295	0.4105	
Inner ring + broken tooth	401	0.0023	0.1720	0.2627	0.4657	0.5695	0.3751	0.2856	5
	402	0.0022	0.1697	0.2400	0.4156	0.5161	0.4363	0.2883	
	403	0.0022	0.1186	0.2662	0.4677	0.5982	0.5006	0.2448	

**Table 6:** Accuracy of different fault features.

Decomposition Method	Inner Ring Fault	Outer Ring Fault	Rolling Body Fault	Inner Ring + Broken Tooth Fault	Inner Ring + Gear Wear Fault	Average Accuracy
EEMD-PSO-MFE	93.3%	97.0%	47.0%	66.7%	66.7%	73.3%
LMD-PSO-MFE	93.3%	90.0%	83.3%	76.7%	83.3%	85.3%
AVMD-FE	100%	100%	90.0%	40.0%	76.7%	81.3%
AVMD-MFE	100%	100%	90.0%	73.3%	80.0%	89.3%
AVMD-PSO-MFE	100%	100%	100%	93.3%	96.7%	98%

## Data Availability

Not applicable.
